# The durable wheat disease resistance gene *Lr34* confers common rust and northern corn leaf blight resistance in maize

**DOI:** 10.1111/pbi.12647

**Published:** 2016-11-15

**Authors:** Justine Sucher, Rainer Boni, Ping Yang, Peter Rogowsky, Heike Büchner, Christine Kastner, Jochen Kumlehn, Simon G. Krattinger, Beat Keller

**Affiliations:** ^1^ Institute of Plant and Microbial BiologyUniversity of ZurichZurichSwitzerland; ^2^ ENSUMR 5667‐ RDPLyonFrance; ^3^ Leibniz Institute of Plant Genetics and Crop Plant Research (IPK)GaterslebenGermany

**Keywords:** durable disease resistance, fungal pathogens, *Lr34/Yr18/Sr57/Pm38*, Maize, common rust, northern corn leaf blight

## Abstract

Maize (corn) is one of the most widely grown cereal crops globally. Fungal diseases of maize cause significant economic damage by reducing maize yields and by increasing input costs for disease management. The most sustainable control of maize diseases is through the release and planting of maize cultivars with durable disease resistance. The wheat gene *Lr34* provides durable and partial field resistance against multiple fungal diseases of wheat, including three wheat rust pathogens and wheat powdery mildew. Because of its unique qualities, *Lr34* became a cornerstone in many wheat disease resistance programmes. The *Lr34* resistance is encoded by a rare variant of an ATP‐binding cassette (ABC) transporter that evolved after wheat domestication. An *Lr34*‐like disease resistance phenotype has not been reported in other cereal species, including maize. Here, we transformed the *Lr34* resistance gene into the maize hybrid Hi‐II. *Lr34*‐expressing maize plants showed increased resistance against the biotrophic fungal disease common rust and the hemi‐biotrophic disease northern corn leaf blight. Furthermore, the *Lr34*‐expressing maize plants developed a late leaf tip necrosis phenotype, without negative impact on plant growth. With this and previous reports, it could be shown that *Lr34* is effective against various biotrophic and hemi‐biotrophic diseases that collectively parasitize all major cereal crop species.

## Introduction

Maize (corn) is one of the most widely grown crops, and maize production passed the mark of 1000 million metric tons in 2013 (FAO, 2016; de Lange *et al*., 2014). Maize is used for human consumption, as a major source of animal feed (Chaudhary *et al*., 2014) and for the production of a number of industrial products such as starch, oil, ethanol and malt. Maize is grown in more than 160 countries over a wide range of agro‐climatic zones.

Fungal diseases are a major threat to maize production, resulting in reduced yields and high input costs for disease control. Global maize yield losses caused by diseases were estimated at about 9% on average in 2001–2003 (Oerke, 2006). Sixty‐two maize‐infecting diseases have been described, and among them, sixteen have been identified as major constraints for maize production (Chaudhary *et al*., 2014). The hemi‐biotrophic fungal disease northern corn leaf blight (NCLB), also known as Turcicum leaf blight (*Exserohilum turcicum*), and the maize common rust disease caused by the biotrophic fungus *Puccinia sorghi* are among these most important foliar diseases (Munkvold and White, 2016). Biotrophic and hemi‐biotrophic fungi have different lifestyles. Obligate biotrophs such as rusts and powdery mildews exclusively grow and reproduce on living plant tissue (Bettgenhaeuser *et al*., 2014; Eckardt, 2006). In contrast, hemi‐biotrophic fungi feed on dead leaf tissue after an initial biotrophic phase that can last from a few hours to several days.

The release of cereal cultivars with high levels of durable field resistance is one of the most effective and sustainable strategies to control fungal diseases (McDonald and Linde, 2002). Hundreds of fungal disease resistance genes have been described in various cereal species (Singla and Krattinger, 2016; Wisser *et al*., 2006). Fungal disease resistance genes can be broadly classified based on their specificity and durability. Race‐specific resistance (*R*) genes are only effective against some isolates of a pathogen, whereas race nonspecific resistance is effective against all pathogen isolates. The former is often controlled by a single major *R* gene encoding for plasma membrane‐localized or intracellular immune receptors, and the latter is often the result of the additive action of several quantitative resistance genes with minor phenotypic effects (Krattinger and Keller, 2016). The deployment of single *R* genes in one cultivar often results in breakdown of disease resistance because of rapid pathogen adaptation. The combination of several race‐specific *R* genes in one cultivar, the use of multiline cultivars with different resistance gene combinations and the use of more durable resistance sources are effective strategies to increase the durability of disease resistance in the field (Brunner *et al*., 2012).

In hexaploid bread wheat (*Triticum asestivum*), three genes have been described that confer race nonspecific resistance against multiple fungal diseases. These three genes, named *Lr34 (=Yr18/Sr57/Pm38), Lr67 (=Yr46/Sr55/Pm46)* and *Lr46 (=Yr29/Sr58/Pm39),* confer partial resistance against all races of the fungal diseases caused by wheat leaf rust (*Puccinia triticina*), stripe rust (*P. striiformis* f.sp. *tritici*), stem rust (*P. graminis* f.sp. *tritici*) and powdery mildew (*Blumeria graminis* f.sp. *tritici*) (Ellis *et al*., 2014; Spielmeyer *et al*., 2013). In addition, these genes are associated with a senescence‐like phenotype referred to as leaf tip necrosis (LTN) (Krattinger *et al*., 2016; Risk *et al*., 2012). In wheat, LTN develops in the flag leaves at the heading stage under some environmental conditions (Singh and Huerta‐Espino, 1997). Two of these genes, *Lr67* and *Lr34,* have been cloned and, respectively, encode a hexose transporter (Moore *et al*., 2015) and a putative ATP‐binding cassette (ABC) transporter (Dakouri *et al*., 2010; Krattinger *et al*., 2009). ABC transporters utilize ATP hydrolysis to translocate substrates across cellular membranes (Jasinski *et al*., 2003; Rea, 2007).

Multiple allelic variants have been found for *Lr34* and *Lr67,* but only one resistance‐conferring variant referred to as *Lr34res* and *Lr67res* has been found for each gene, respectively. Interestingly, both *Lr34res* and *Lr67res* are the result of rare mutation events that occurred after domestication in ancient wheat landraces (Kolmer *et al*., 2008; Krattinger *et al*., 2013; Moore *et al*., 2015). The LR34res protein version differs by only two amino acid polymorphisms from the LR34sus protein of which one is critical for disease resistance (Chauhan *et al*., 2015; Krattinger *et al*., 2009). Bread wheat was domesticated around 10 000 years ago, long after wheat shared its last common ancestor with other cereal species. An *Lr34*‐like disease resistance has so far not been described in other globally important cereals like rice and maize. *Lr34res* was introduced into modern wheat in the 1900s by the Italian wheat breeder Nazareno Strampelli and has since been extensively used for crop protection (Kolmer *et al*., 2008). In particular, the International Maize and Wheat Improvement Center (CIMMYT) continues to use combinations of partial rust resistance genes with additive effects in its rust improvement programme (Singh *et al*., 1991, 2005).

Orthologous *Lr34* genes are present in rice and sorghum, but not in maize and in barley (Krattinger *et al*., 2011, 2013). However, only the susceptible haplotype has been found among these *Lr34* orthologs which is in agreement with the very recent emergence of the *Lr34res* allele after wheat domestication. Despite the absence of the resistant *Lr34* haplotype in cereals other than wheat, *Lr34res* was functionally transferred into barley and rice, where it provides resistance against the adapted barley pathogens barley leaf rust (P*uccinia hordei*) and barley powdery mildew (*Blumeria graminis* f.sp. *hordei*) as well as the rice‐specific fungus rice blast (*Magnaporthe oryzae*), respectively (Krattinger *et al*., 2016; Risk *et al*., 2013). In barley, the expression of *Lr34res* resulted in high levels of disease resistance at the seedling stage. This was, however, accompanied by a strong LTN phenotype which had a negative impact on plant growth and yield (Chauhan *et al*., 2015; Risk *et al*., 2013). In rice, one of the transgenic lines showed a late LTN phenotype resulting in a plant development similar to the nontransgenic sib line but increased levels of rice blast resistance (Krattinger *et al*., 2016).

In this study, we transformed the Hi‐II hybrid maize line with *Lr34res* driven by its native promoter. *Lr34res*‐expressing maize lines showed an increased resistance against two of the most important foliar maize diseases: common rust and northern corn leaf blight.

## Results

### *Lr34res* confers common rust and northern corn leaf blight resistance in maize

Thirteen fertile independent primary transgenic (T0) plants were generated in the maize hybrid Hi‐II by Agrobacterium‐mediated transformation. DNA blot analyses indicated that these 13 plants carry at least two insertions of the *Lr34res* transgene (one line had two copies, seven lines had three copies, and five lines had more than three copies).

Three independent transgenic events, called 161, 163 and 164, respectively, were selected for further characterization. Each of them carried three co‐segregating copies of *Lr34res* (Figure S1b), and they were chosen based on the development of a LTN phenotype and on the number of available T1 kernels. *Lr34res* and sib T1 plants were advanced to the T2 generation and homozygous T2 plants for events 163 and 164, and segregating T2 plants for event 161 were selected for further analyses. For event 161, all plants were analysed by PCR for the presence or absence of *Lr34res*.

**Figure 1:**
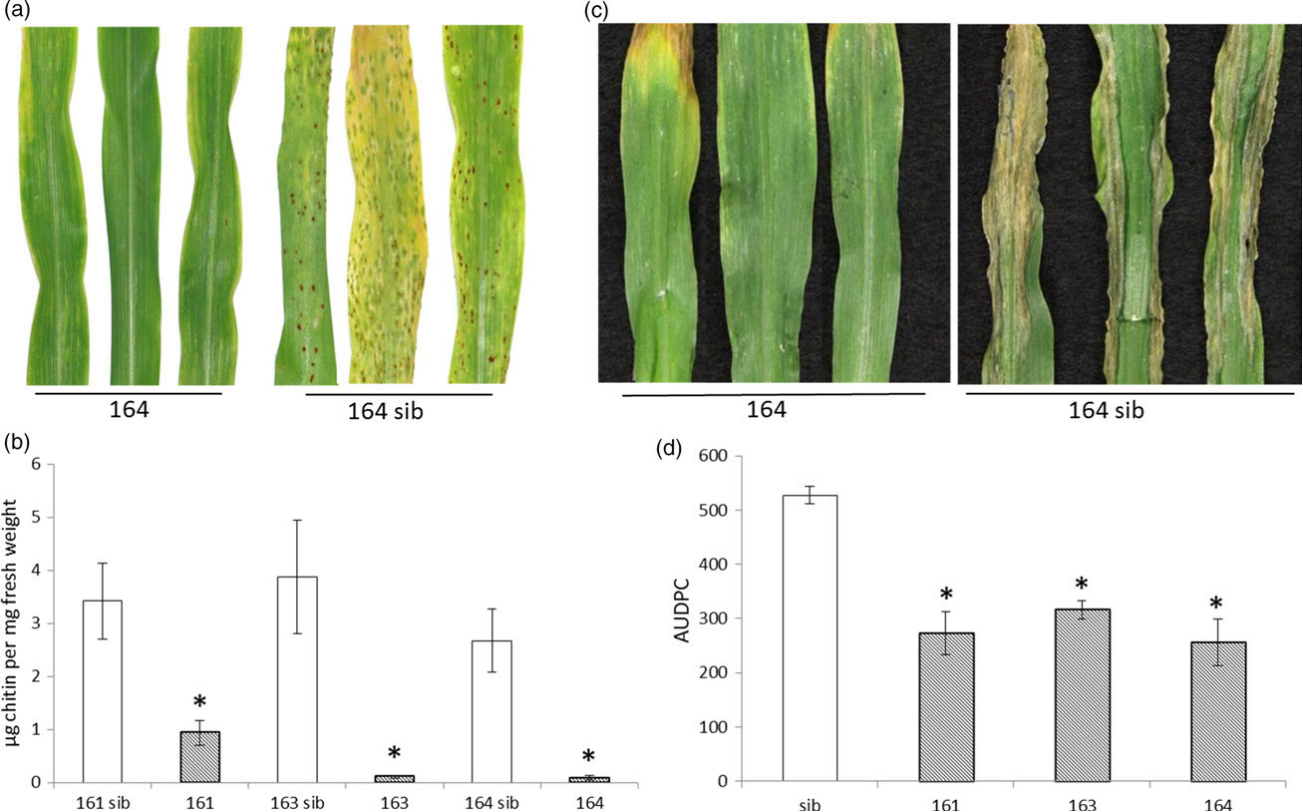
*Lr34res* provides partial resistance against common rust and NCLB in maize. (a) Macroscopic evaluation of common rust symptoms on seedling leaves of *Lr34res* transgenic maize event 164 and corresponding sib plant 12 days after infection. (b) WGA‐FITC chitin quantification of common rust, 12 d.a.i. * indicates significant differences between *Lr34res*‐expressing plant and sibs (Mann–Whitney–Wilcoxon test *W* = 25*, P* < 0.05). Error bars represent standard errors of five biological replicates. (c) Macroscopic observation of NCLB symptoms ten d.a.i on *Lr34res* event 164 and its corresponding sib line. (d) Area under the disease progress curve (AUDPC) of the different *Lr34res* transgenic individuals and sibs (pool of sib derived from the different events) calculated between day 8 to day 13 after infection. Error bars represent standard errors from at least three independent experiments. * indicates a significant differences to the sibs (Mann–Whitney–Wilcoxon test, *W* = 18 or 24*, P* < 0.05).

Pathogenicity analyses were undertaken on these three independent T2 families using the biotrophic fungus common rust and the hemi‐biotrophic fungus NCLB. For common rust, all three lines showed markedly increased resistance compared to their sibs lacking *Lr34res*. The plants derived from events 163 and 164 did not develop macroscopic symptoms 12 days after infection (d.a.i.), whereas their sibs showed brownish pustules typical for common rust. A few and smaller pustules were visible on *Lr34res*‐expressing plants of event 161 (Figure 1a, Figure S2). We quantified fungal biomass in infected plant tissue using fluorescently labelled wheat germ agglutinin (WGA‐FITC) that specifically binds to the fungal cell wall component chitin (Ayliffe *et al*., 2013). Similar to the macroscopic observations, the *Lr34res*‐expressing maize plants of all three independent events analysed in detail had significantly reduced chitin levels compared to their sibs (Figure 1b). Chitin levels in event 161 were higher (0.93 μg/mg fresh weight) compared to plants of events 163 (0.10 μg) and 164 (0.089 μg), which is in agreement with the weaker macroscopic resistance phenotype in the former. However, even 161‐derived transgenics had much reduced chitin levels compared with their sibs lacking *Lr34res* (2.67–3.41 μg).

NCLB symptoms were evaluated macroscopically ten and 14 d.a.i. *Lr34res‐*expressing maize plants showed a delay in symptom development with smaller lesions compared to the sibs. At ten d.a.i., most of the *Lr34res* plants did not show visible signs of infection, whereas most of the sib plants already showed severe symptoms (Figure 1c). After 14 days, symptoms also appeared on *Lr34res* plants, but they were less severe than on sib lines (Figure S3). Disease severity was also quantitatively assessed by scoring the percentage of plants showing symptoms from eight to 13 d.a.i. and the area under the disease progress curve (AUDPC) was calculated according to Hurni *et al*. (2015). This quantification again revealed a delay in NCLB development in plants of the three *Lr34res*‐expressing T2 families (Figure 1d). Our infection experiments showed that *Lr34res* confers a reduction in *P. sorghi* and *E. turcicum* development. The partial resistance phenotype is reminiscent of *Lr34res*’ partial resistance phenotype in wheat.

### *Lr34res* expression in maize results in LTN but no apparent growth reduction

**Figure 2:**
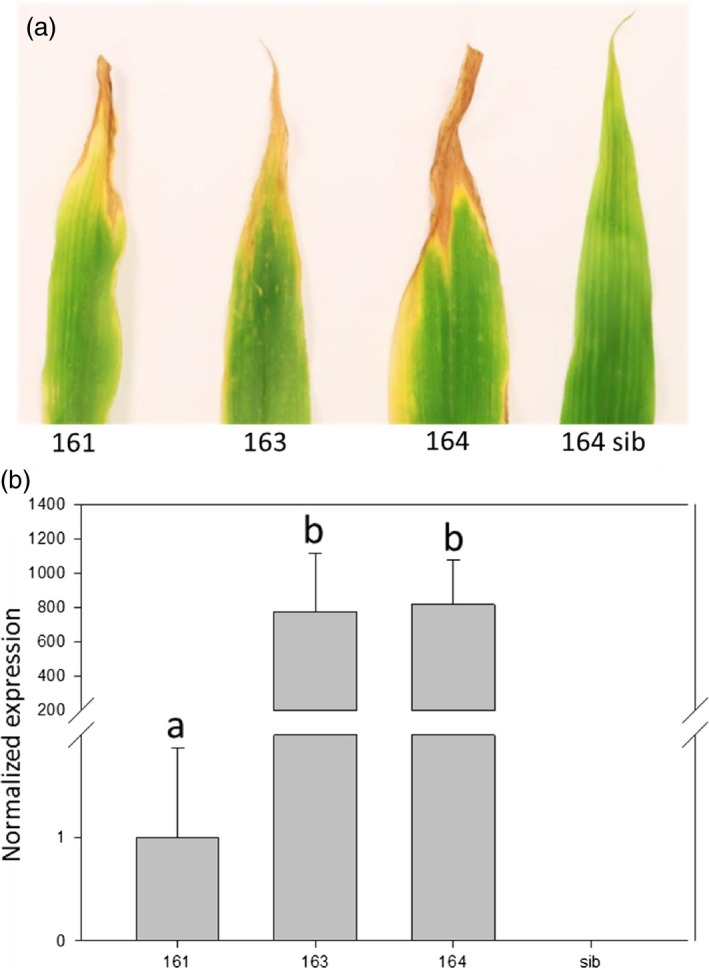
Expression of *Lr34res* results in a LTN phenotype in the three transgenic lines. (a) Representative example of the third leaf of six‐week‐old seedlings showing a characteristic LTN phenotype. (b) Normalized *Lr34res* expression in transgenic lines. Data are normalized to the reference gene folylpolyglutamate synthase (FPGS). *Lr34res* expression level of line 161 = 1. Letters indicate line with equivalent expression level (Mann–Whitney–Wilcoxon *test, P* < 0.05). Errors bars represent standard errors from three biological replicates.

In barley and in rice, high *Lr34res* expression levels at the seedling stage were associated with a strong LTN phenotype resulting in a negative impact on plant development (Krattinger *et al*., 2016; Risk *et al*., 2013). Among four transgenic rice events, one showed low *Lr34res* seedling expression levels coupled with late LTN development and no obvious growth penalty (Krattinger *et al*., 2016). All three transgenic maize events developed a LTN phenotype appearing after ~6 weeks (Figure 2a). At the seedling stage, when the expression level was measured and the infection tests were performed, no LTN was visible. Hence, the *Lr34res*‐based resistance was apparent before LTN emerged.

**Figure 3:**
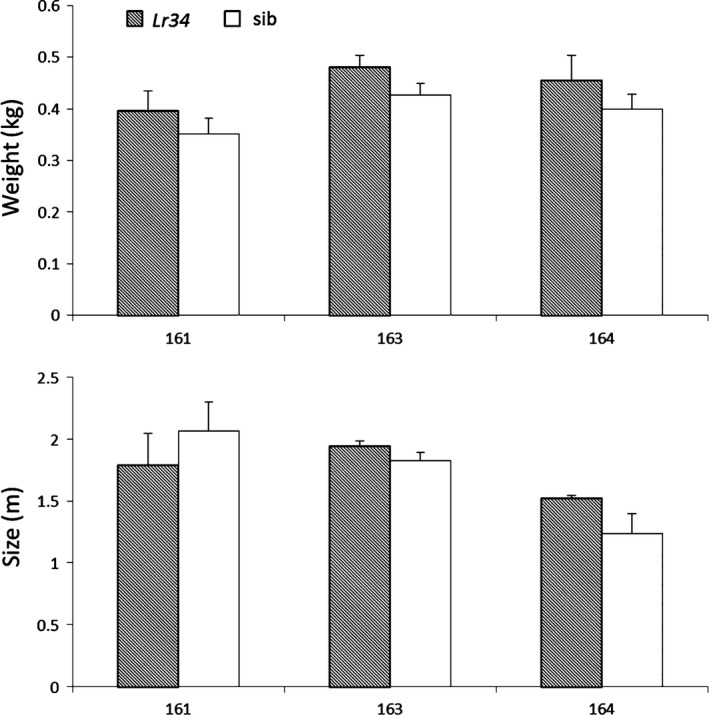
Measurement of growth parameters in *Lr34res* maize plants. Plant fresh weight in kg and plant height in m was assessed for the different plants and corresponding sibs using at least three biological replicates of 14‐week‐old plants. Statistical analysis was performed using the Mann–Whitney–Wilcoxon test and was always *P* < 0.05. Errors bars represent standard errors from the three to seven biological replicates.

Seedling expression levels of the three independent maize events were assessed by RT‐qPCR. Interestingly, plants derived from events 163 and 164 had 774‐fold and 816‐fold higher *Lr34res* expression levels compared to those of event 161 (Figure 2b). As shown above, despite the relatively low *Lr34res* expression levels in event 161, it showed a clear NCLB and common rust resistance phenotype. To determine a possible negative impact of *Lr34res* on plant development, we measured two parameters: plant height and aboveground fresh weight of *Lr34res*‐expressing maize plants and respective sibs grown under glasshouse conditions (Figure 3). These parameters appear to be good indicators of potential negative impacts of *Lr34res* on plant development as they show an obvious reduction in *Lr34res*‐expressing barley plants and the high‐expressing rice lines (Krattinger *et al*., 2016; Risk *et al*., 2013). In *Lr34res*‐expressing maize plants, however, no significant differences were observed for these parameters compared with the respective sib lines. Also, visually there was no apparent impact on the plant development when *Lr34res* was expressed. These results indicate that unlike barley and rice, even a relatively strong expression of *Lr34res* as found in events 163 and 164 does not impact plant growth under our growth conditions.

### Orthologous *Lr34* genes in maize and distant relatives

Based on a comparative analysis, Krattinger *et al*. (2011) showed that the most similar maize homologue of the LR34 protein, GRMZM2G014282, was only 58% identical to LR34res. In contrast, rice and sorghum have more similar LR34 orthologs with 86% and 72% amino acid identity, respectively. To test for the presence of *Lr34* orthologs in the *Zea* lineage (maize) and its sister taxa, the *Tripsacum* lineage, a PCR probe was developed based on sequences that are conserved among *Lr34* sequences of wheat, rice and sorghum. This PCR probe spanned the critical sequence polymorphisms that distinguish *Lr34res* from *Lr34sus*. PCR was performed on genomic DNA of bread wheat (cv. Chinese Spring), rice (cv. Nipponbare), maize (Hi‐II) and ten different *Tripsacum* species (Table S1). Wheat, rice and *Tripsacum* showed PCR amplification of the expected fragment size, but no amplification product was produced from maize (Figure S4a). The amplification products of *Tripsacum* were sequenced and showed greater than 72% nucleotide identity compared to the *Lr34* wheat sequence. The corresponding fragment of the rice and sorghum orthologs, *OsABCG50* and *Sb01 g016775,* showed 90% and 77% nucleotide identity, respectively, whereas no significant blast of this segment was obtained against the maize homologue GRMZM2G014282. All the *Tripsacum* sequences clustered within the ‘*Lr34* clade’ in a phylogenic tree (Figure S4b), and all the *Tripsacum* species had the susceptible haplotype for the two critical sequence polymorphisms.

## Discussion

In this study, we showed that the wheat *Lr34res* allele is functionally transferable into maize and confers resistance against two maize diseases of agronomic importance: the biotrophic common maize rust pathogen and the hemi‐biotrophic fungus that causes northern corn leaf blight disease. Hence, *Lr34res* can be effective against biotrophic and hemi‐biotrophic diseases in the same crop species. *Lr34res* has already been shown to be effective against the hemi‐biotrophic fungus rice blast in rice (Krattinger *et al*., 2016) and various biotrophic fungi such as rust and powdery mildew pathogens in wheat and barley (Risk *et al*., 2012, 2013). Furthermore, *Lr34res* has been associated with resistance against the hemi‐biotrophic fungus spot blotch (*Bipolaros sorokiniana*) in wheat (Lillemo *et al*., 2013).

The infection processes of these fungi involve several steps, including prepenetration, penetration, colonization and sporulation (Tucker and Talbot, 2001). Each fungus uses a different mechanism to invade its host plant. Penetration can be direct, through the cuticle and the different layers of the cell wall, as this is the case for rice blast, NCLB and powdery mildew pathogens (Haugaard *et al*., 2002; Knox‐Davies, 1974; Pederson *et al*., 2012; Wathaneeyawech *et al*., 2015; Zabka *et al*., 2008; Zhang *et al*., 2014). Other pathogens like the rusts use natural openings such as stomata on the leaf surface (Song *et al*., 2011). Once inside the leaf, the obligate biotrophic rust and mildew fungi produce specialized feeding structures called haustoria for nutrient uptake and effector secretion (Haugaard *et al*., 2002; Pederson *et al*., 2012; Petre and Kamoun, 2014; Song *et al*., 2011; Zabka *et al*., 2008). The hemi‐biotrophic fungi rice blast and NCLB also develop vesicles‐like structures by differentiation of primary hyphae (Kankanala *et al*., 2007; Muiru *et al*., 2008; Wilson *et al*., 2012) that are very similar to haustoria and that are also surrounded by the host membrane (Levy and Cohen, 1983; Wilson and Talbot, 2009). In contrast to the biotrophic pathogens, however, rice blast and NCLB switch to a necrotrophic growth phase after about two to three days (Levy and Cohen, 1983; Marcel *et al*., 2010). The biotrophic growth phase is a commonality among all pathogens *Lr34res* is effective against, and although the exact molecular function of this gene is still unknown, it is likely that the LR34res protein restricts fungal growth during this phase.

Qualitative and quantitative NCLB resistance genes have been identified in maize, for example the race‐specific *Ht1* gene (Welz and Geiger, 2000) and the quantitative gene *Htn1* that confers resistance against the most prevalent NCLB races (Hurni *et al*., 2015). *Htn1* encodes a putative wall‐associated receptor‐like kinase that might be involved in the perception of an apoplastic signature of *E. turcicum* (Hurni *et al*., 2015). Common rust resistance genes have been mapped to eight different loci (Rp1, 3, 4, 5, 6, 7, 8 and 9) (Hulbert, 1997). *Rp1* and *Rp3* are the best characterized loci, and each consists of a cluster of genes that encode intracellular nucleotide‐binding, leucine‐rich repeat receptor (NLR) proteins (Smith *et al*., 2010; Webb *et al*., 2002). Gene number at the *Rp1* locus differs considerably among different maize cultivars and can vary from one gene in some haplotypes to more than 50 paralogous genes in others (Smith *et al*., 2004). Within four maize lines (*HRp1*‐*B*,* HRp1*‐*M*,* MH95* and *B73*), a total of 61 *Rp1* genes, with a large diversity in the LRR region, were identified. Among them, thirty‐two were transcribed and each of these genes can be distinguished based on the *P. sorghi* isolate to which it confers resistance (Chavan *et al*., 2015). An example is the *Rp1*‐*D* gene, which has been successfully used in North America between 1985 and 2000. During autumn 1999, however, the emergence of a new *Rp1‐D*‐virulent rust population infected more than 40% of the *Rp1‐D* ‘resistant hybrids’ (Pataky and Pate, 2001). This demonstrates that disease resistance based on single NLR genes is prone to breakdown because of the emergence and rapid spread of new pathogen strains. In contrast, *Lr34res* is a durable resistance gene with no pathogen adaptation observed so far in wheat. It is therefore possible that the resistance observed in maize against common rust and NCLB will also be durable. Hence, this gene might be useful in maize disease resistance breeding.

Orthologs of *Lr34* are found in some cereals, including rice with OsABCG50 (86% amino acid identity) and sorghum with Sb01g016775 (74% amino acid identity) and Sb01g016770 (72% amino acid identity) (Krattinger *et al*., 2011). Members of the grass genus *Tripsacum,* a distant relative of *Zea,* also carry *an Lr34* ortholog. Maize does not have an ortholog of *Lr34* but can be crossed with *Tripsacum*. As shown by James in 1979, crosses between maize and various *Tripsacum* species result in pollen‐sterile hybrids but produce kernels after backcrossing with maize. Maize × *Tripsacum* hybrids were already used in maize breeding programmes for the introgression of traits of interest, like fatty acid composition (Duvick *et al*., 2006) or rootworm resistance (Prischmann *et al*., 2009). The *Tripsacum* accessions tested in this study all had the susceptible *Lr34* haplotype. However, *Lr34res* in wheat was the result of rare mutation events. Hence, it is possible that *Lr34* orthologs of the resistant haplotype might be found in a large *Tripsacum* collection at low frequency. Such rare variants could be crossed into the modern maize genepool by *Tripsacum* × maize hybridizations. If no resistant *Lr34* variants can be found, genome editing could be used to transform the susceptible into resistant haplotype.

Interspecies gene transfer is a very powerful tool to move resistances between species and can thereby result in a broader resistance spectrum. For example, the NLR gene *Rxo1* has been successfully transferred from maize, where it provides resistance against diverse bacterial pathogens, such as *Xanthomonas oryzae* pv. *oryzicola*, the causal agent of bacterial streak disease, to rice (Zhao *et al*., 2005). Transgenic wheat expressing the Arabidopsis EFR receptor‐like kinase was more resistant against the bacterial disease *Pseudomonas syringe* pv. *oryzae*. EFR perceives the highly conserved bacterial elongation factor Ef‐Tu (Schoonbeck *et al*., 2015). Similarly, the rice receptor‐like kinase (RLK) gene *Xa21* has been shown to provide Banana Xanthomonas wilt resistance in banana (Tripathi *et al*. 2014). The nonhost resistance NHR‐linked Arabidopsis genes *PING 4* (coding for a phospholipase‐like protein (EARLI4‐like)), *PING* 5 (coding for a leucine‐rich repeat protein kinase) and *PING* 6 (coding for an ankyrin repeat family protein), known to provide pre‐invasion resistance to nonadapted fungal pathogens, have been transferred into soya bean where they confer Asian soya bean rust resistance (Langenbach *et al*., 2016). *Lr34res* also has been successfully transferred in other crop species such as barley and rice (Krattinger *et al*., 2016; Risk *et al*., 2013). The fact that this gene functions across different monocots indicates that the mechanism of resistance is conserved and that the substrate transported by the ABC transporter protein is likely shared among them.

It is known that expression of resistance genes often results in lower shoot biomass and negative effects (Burdon and Thrall, 2003). *Lr34res* is also known to negatively impact plant development in barley (Chauhan *et al*., 2015; Risk *et al*., 2013) and in rice, where it was, however, possible to balance the fitness cost by modulating the gene expression level at seedling stage (Krattinger *et al*., 2016). From our glasshouse experiments, even under high *Lr34res* expression levels, the development of *Lr34res*‐expressing maize plants was not negatively affected. Whether *Lr34res* in maize does result in a small yield penalty or not can only be determined in field experiments.

To conclude, these results are a proof of concept that *Lr34res* is functionally transferrable into the globally most produced cereal crop, maize. Hence, this resistance gene is functional in all major cereals tested. In contrast to wheat and rice, however, transgenic maize plants are already commercially grown and more than 30% of the maize area was planted with GM plants in 2014 (Lucht, 2015). Besides a transgenic approach, a detailed analysis of the *Lr34* ortholog in *Tripsacum* could offer a nontransgenic approach to establish *Lr34*‐like disease resistance in maize.

## Experimental procedures

### Maize transformation and plant characterization

The genomic *Lr34res* construct under the wheat native promoter and terminator in the binary vector p6U (*p6U:gLr34res*; Risk *et al*., 2012) was transformed into the maize Hi‐II (A188 x B73) hybrid (Horn *et al*., 2006) using *Agrobacterium tumefaciens*‐mediated transformation according to a protocol previously established (Hensel *et al*., 2009; Van der Linde *et al*., 2012). Fifty‐five T0‐plants were recovered and the identification of the *Lr34res* transformants was done by PCR on genomic DNA using the *cssfr1* marker (Lagudah *et al*., 2009). The copy number was assessed by Southern blot using 7 μg of *Eco*RI‐digested genomic DNA and a *hygromycin phosphotransferase* (Ubi‐HPT) ^32^P‐labelled probe (Figure S1a) (Risk *et al*., 2013). Plants were self‐pollinated, resulting in T1 kernels.

### Maize rust infection

Three‐week‐old maize seedlings were grown in Jiffy pots (diameter 8 cm) with standard soil (Classic ED 73) in a growth cabinet under diurnal conditions with 16 h light at 20 °C and 8 h dark at 18 °C. Plants were then spray‐infected with a *P. sorghi* isolate that was collected from a natural glasshouse infection on maize cultivar *sweet nugget*. Plants were sprayed using an air‐assisted sprayer, with a solution of urediniospores (incubated at 40 °C for five minutes to break the dormancy) and oil (Fluorinert FC‐43). After inoculation, infected plants were incubated in the dark under high humidity (~95%) at 16 °C for 24 h. Then, plants were shifted to diurnal conditions (16‐h light/20 °C, 8‐h dark/16 °C, 70% humidity). Disease symptoms were assessed macroscopically for the presence of pustules on the leave surface and using chitin quantification 12 days after infection. Quantification of chitin was performed as described in Ayliffe *et al*. (2013). Five biological replicates of plant leaf tissue were harvested, weighed, cut into 3‐cm fragments and placed into 50‐mL Falcon tubes. Sufficient volume of 1 m KOH containing 0.1% (vol/vol) Silwet L‐77 (Lehle Seeds, Round Rock, TX) was added to each tube to entirely cover the tissues. Tissues were then heated in a steam cooker for 20 min before being washed with 50 mL of 50 mm Tris, pH 7.0 and resuspended in a 50 mL volume of Tris. Plant tissues were macerated by sonication to generate a fine and uniform suspension. Two‐hundred microlitre of 1 into ten water‐diluted samples were added to 10 μL of a 1‐mg/mL solution of WGA‐FITC before being measured using the fluorometer Synergy H1 Hybrid Reader (Biotek). The read‐outs were analysed using the software Gen5, version 2.03.1 (Biotek).

### NCLB cultivation and infection

*Exserohilum turcicum* isolate passau‐1 described by Hurni *et al*., 2015 was grown *in vitro* for three weeks on potato dextrose agar (PDA) (19.5 g/500 mL of ddH_2_O) plates in the dark, upside down, at room temperature. Spores were harvested from plates by rinsing with 0.1% Tween 20 (Carl Roth) in sterile water and scraped with a spatula and filtered through a fine mesh (0.5 mm). Final density was adjusted to 4.5 * 10^4^ spores per mL, using a Neubauer counting chamber and sprayed on the three‐week‐old plants. Maize plants were grown for three weeks in Jiffy pots with standard soil (Classic ED 73) (Einheitserde), 15 plants in the same tray, in the glasshouse with cycles of 16 h at 20 °C with light and 8 h at 18 °C in the dark. Once the second leaf was fully expanded, the newly emerged young leaves were removed by cutting until the end of the experiment as described in Hurni *et al*. (2015).

Infected plants were kept under the same conditions but covered by plastic hoods to maintain a high humidity. First symptoms were observed eight d.a.i. and were then recorded every day for five days. Disease severity was calculated as previously described by Hurni *et al*. (2015) and Madden *et al*. (2007).

### Quantitative real‐time PCR analysis of *Lr34res* expression

Expression of *Lr34res* was quantified using RT‐qPCR. Total RNA was extracted from three‐week‐old seedling leaves using the SV Total RNA Isolation System (Promega). RNA was quantified using a Nanodrop ND‐1000 spectrophotometer, and RNA integrity was checked on a 0.8% agarose gel in 1× sodium‐borate (SB) buffer. The cDNA synthesis was then performed from 500 ng of RNA using the iScript™ advanced cDNA synthesis kit (Bio‐Rad).

Expression of *Lr34res* was determined by RT‐qPCR in a 10‐μL volume reaction including 5 μL of KAPA SYBR^®^ fast qPCR master mix (KAPA Biosystems), forward and reverse *Lr34res* qPCR primer (Risk *et al*., 2012) (500 nm final concentration) and 4 μL of 1:20 diluted cDNA. Samples were run on a CFX96 Touch Real‐time PCR (Bio‐Rad). Folylpolyglutamate synthase (FPGS) was used as reference gene with the primers Forward 5′ ATCTCGTTGGGGATGTCTTG 3′ Reverse 5′ AGCACCGTTCAAATGTCTCC 3′ (Manoli *et al*., 2012).

### Determination of growth parameters under glasshouse conditions

At least three plants per transformation event were kept until adult stage (14 weeks) in a glasshouse under LED light. The glasshouse conditions were 60% humidity and 25 °C. Plants were grown in standard soil (Classic ED 73) (Einheitserde). Fourteen‐week‐old plants were cut at soil level to determine fresh weight and plant height to the apex. Tassels were cut at their base and therefore excluded from the measure.

### Identification of *Lr34* orthologous genes in maize and *Tripsacum*

PCR was performed on genomic DNA using primer set *Lr34_conserved_f2* (5′‐CCATGGCCCTCGAAATGAAG‐3′) and *Lr34_conserved_r2* (5′‐GCTGGTATTGCATATGCCCA‐3′).

PCRs were performed in a 20‐μL total volume using 1 μL of 40 ng/μL gDNA and 19 μL of PCR mix (Sigma buffer 1× (Sigma–Aldrich), dNTPs (0.125 mm), primer forward and reverse (0.5 mm) and Taq polymerase (Sigma–Aldrich) (0.25 μL of 5 U/μL)).

PCR products (~755 bp) were purified using the GenElute Gel extraction kit (Sigma–Aldrich). Purified products were sequenced using 1 μL of purified PCR product, 0.80 μL Big Dye/BrightDye Terminator Mix (Thermo‐Fisher), 0.25 μL primer (5 pmol/μL) and 2 μL of 5× sequencing buffer in a total reaction volume of 10 μL. Sequencing was performed using an ABI‐3730 Sanger sequencer (Applied Biosystems) and sequences were analysed with FinchTV^®^ software (Geospiza).

Sequence alignments and phylogenetic tree construction were performed using the web service phylogeny.fr (Dereeper *et al*., 2008, 2010). The multiple alignment was performed with MUSCLE^®^ software (Edgar, 2004), the alignment curation with GBlock^®^ software (Castresana, 2000; Talavera and Castresana, 2007), the construction of the phylogenetic tree with PhyML^®^ (Guindon *et al*., 2010) and the tree rendering with TreeDyn^®^ (Chevenet *et al*., 2006).

## Conflict of interest statement

The authors declare that the research was conducted in the absence of any commercial or financial relationship that could be construed as a potential conflict of interest.

## Supporting information


**Figure S1.** Transgene copy number detection. (a) Schematic representation of the *Lr34res* construct. (b) Southern blot showing T‐DNA copy number in the *Lr34res* transgenic maize. Lines 161, 163 and 164 at T2 generation are represented. + indicates plants with *Lr34res* and – segregating sib lines without *Lr34res*.
**Figure S2.** Macroscopic common rust symptoms on plants derived from events 161 and 163 and corresponding sibs, 12 d.a.i. Infections were done at seedling stage on three‐week‐old plants.
**Figure S3.** Macroscopic observation of NCLB symptoms on the different *Lr34res* transgenic maize plants and their corresponding sibs 14 days after infection. Infections were done at seedling stage on three‐week‐old plants. Scale bar = 10 mm.
**Figure S4.** Study of orthologous *Lr34* genes in maize and its distant relatives of the genus *Tripsacum*. (a) Agarose gel showing genomic DNA amplification of *Lr34* on wheat (Chinese‐Spring), *OsABCG50* on rice (Nipponbare) and the *Lr34* orthologs on 10 different *Tripsacum* species. No amplification was obtained for maize Hi‐II. (b) Phylogenetic tree based on genomic DNA sequences of *Lr34*, of the most homologous maize gene sequence (GRMZM2G014282), of three of the most homologous rice genes sequences (*OsABCG50*,* OsABCG41* and *OsABCG49)*, two sorghum orthologous sequences (*Sb01 g016775* and *Sb01 g016700)*, one *Brachypodium distachyon* homolog (*Bradi4 g45397)*, and 5 different *Tripsacum* species. The “*Lr34* orthologous cluster” is marked in red. The sequence from *Penicillium chrysogenum* (*Pc12 g09900*) was used as outgroup to root the tree (Krattinger *et al*., 2011). Numbers indicate how many times the sequences to the right of the fork occurred in the same group out of 100 trees.
**Table S1.** List of the 13 *Tripsacum* accessions used for the study of the *Lr34* orthologous gene. ID number corresponds to the reference in the CIMMYT maize germplasm database.
