# Supplementary material for: The durable wheat disease resistance gene Lr34 confers common rust and northern corn leaf blight resistance in maize
Source: Plant Biotechnol J. 2016 Nov 15;15(4):489–96. doi: 10.1111/pbi.12647 (PMC5362690; doi:10.1111/pbi.12647)
Supplement: Supplementary file 1 — Figure S1. Transgene copy number detection. (a) Schematic representation of the Lr34res construct. (b) Southern blot showing T‐DNA copy number in the Lr34res transgenic maize. Lines 161, 163 and 164 at T2 generation are represented. + indicates plants with Lr34res and – segregating sib lines without Lr34res. Figure S2. Macroscopic common rust symptoms on plants derived from events 161 and 163 and corresponding sibs, 12 d.a.i. Infections were done at seedling stage on three‐week‐old plants. Figure S3. Macroscopic observation of NCLB symptoms on the different Lr34res transgenic maize plants and their corresponding sibs 14 days after infection. Infections were done at seedling stage on three‐week‐old plants. Scale bar = 10 mm. Figure S4. Study of orthologous Lr34 genes in maize and its distant relatives of the genus Tripsacum. (a) Agarose gel showing genomic DNA amplification of Lr34 on wheat (Chinese‐Spring), OsABCG50 on rice (Nipponbare) and the Lr34 orthologs on 10 different Tripsacum species. No amplification was obtained for maize Hi‐II. (b) Phylogenetic tree based on genomic DNA sequences of Lr34, of the most homologous maize gene sequence (GRMZM2G014282), of three of the most homologous rice genes sequences (OsABCG50, OsABCG41 and OsABCG49), two sorghum orthologous sequences (Sb01 g016775 and Sb01 g016700), one Brachypodium distachyon homolog (Bradi4 g45397), and 5 different Tripsacum species. The “Lr34 orthologous cluster” is marked in red. The sequence from Penicillium chrysogenum (Pc12 g09900) was used as outgroup to root the tree (Krattinger et al., 2011). Numbers indicate how many times the sequences to the right of the fork occurred in the same group out of 100 trees. Table S1. List of the 13 Tripsacum accessions used for the study of the Lr34 orthologous gene. ID number corresponds to the reference in the CIMMYT maize germplasm database. [file PBI-15-489-s001.pdf]

(a)

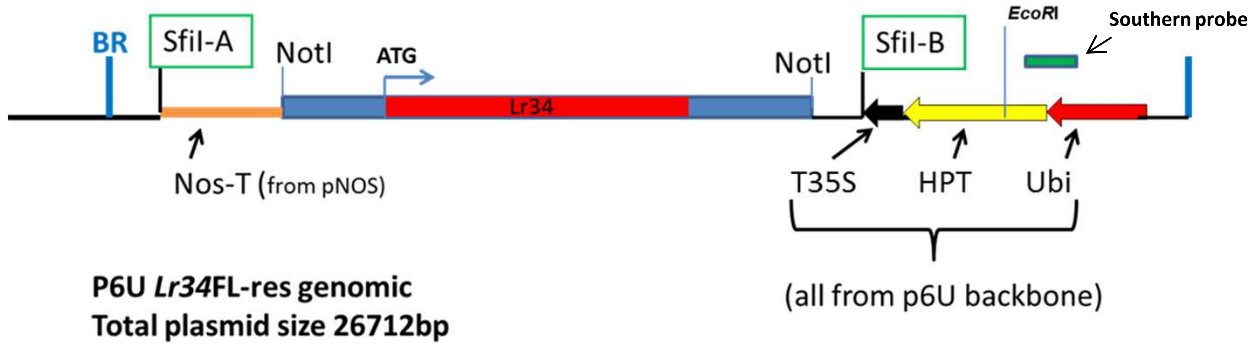

(b)

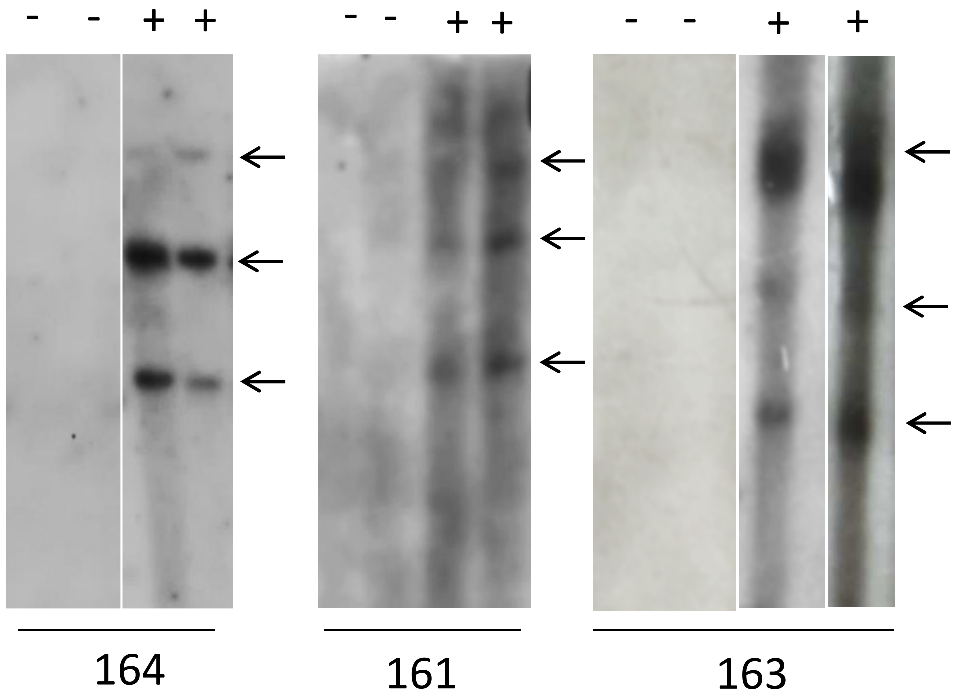

Figure S1. Transgene copy number detection. a) Schematic representation of the *Lr34res* construct. b) Southern blot showing T-DNA copy number in the *Lr34res* transgenic maize. Lines 161, 163 and 164 at T2 generation are represented. + indicates plants with *Lr34res* and – segregating sib lines without *Lr34res*.

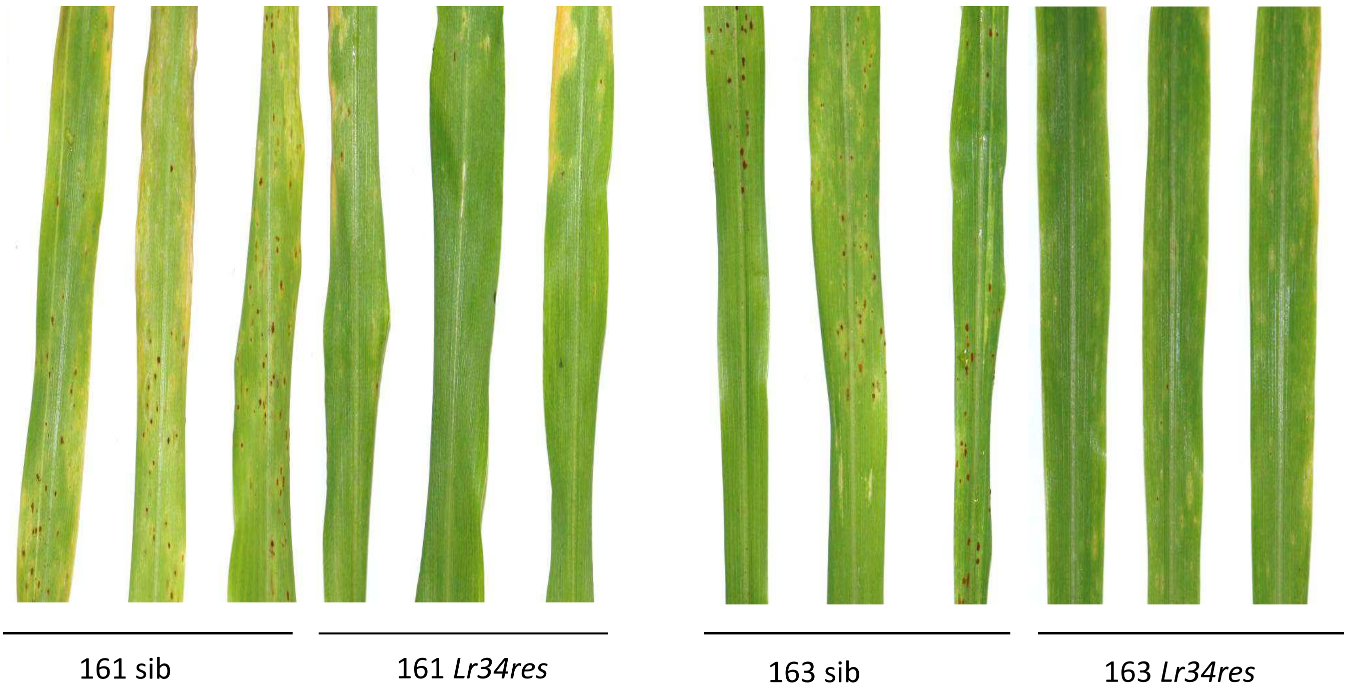

Figure S2. Macroscopic common rust symptoms on plants derived from events 161 and 163 and corresponding sibs, 12 d.a.i. Infections were done at seedling stage on three-week-old plants.

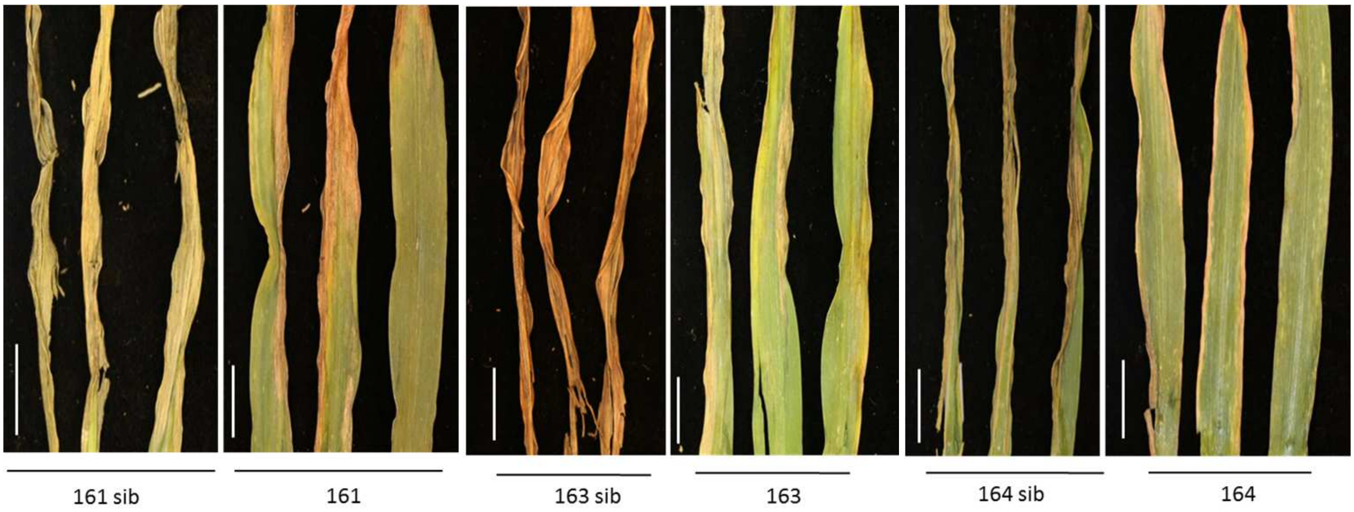

Figure S3. Macroscopic observation of NCLB symptoms on the different *Lr34res* transgenic maize plants and their corresponding sibs 14 days after infection. Infections were done at seedling stage on three-week-old plants. Scale bar = 10 mm.

Table S1. List of the 13 T*ripsacum* accessions used for the study of the *Lr34* orthologous gene, with the name of the different species, their corresponding ID and accession numbers from the CIMMYT maize germplasm database and their country of origin .

Species Name	ID	ACC	Country
dactyloides	28224	25536	USA
dactyloides var.meridonale	28253	25565	VEN
cundinamarce	28254	25566	COL
andersonii	28262	25574	HND
jalapense	28305	25617	MEX
jalapense	28309	25621	MEX
australe	14651	12826	COL
peruvianum	28334	25646	PER
peruvianum	28335	25647	ECU
maizar	28356	25668	MEX
maizar	28357	25669	MEX
pilosum	28358	25670	MEX
lanceolatum	28360	25672	MEX

(a)

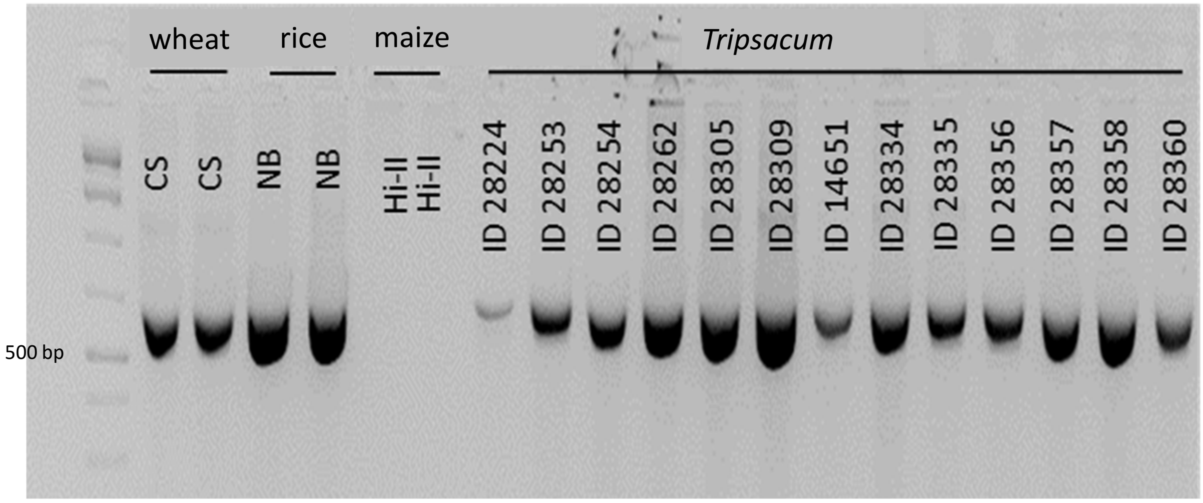

(b)

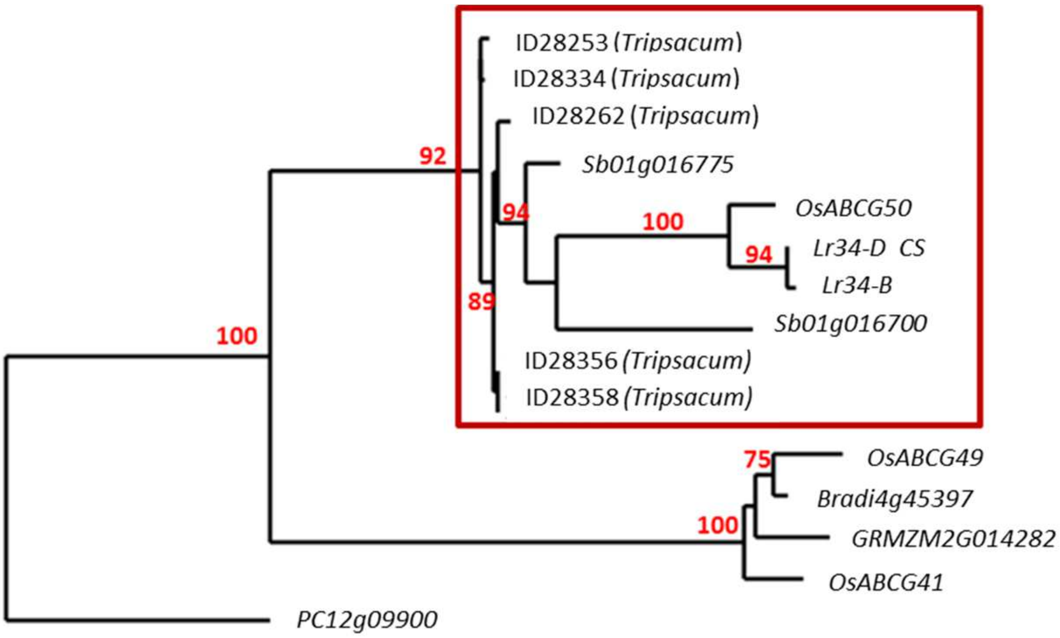

Figure S4. Study of orthologous *Lr34* genes in maize and its distant relative *Tripsacum*. (a) Agarose gel showing genomic DNA amplification of *Lr34* on wheat (Chinese-Spring), *OsABCG50* on rice (Nipponbare) and the *Lr34* orthologs on 10 different *Tripsacum* species. No amplification was obtained for maize Hi-II. (b) Phylogenetic tree based on genomic DNA sequences of *Lr34*, of the most homologous maize gene sequence (GRMZM2G014282), three of the most homologous rice genes sequences (*OsABCG50*, *OsABCG41* and *OsABCG49)*, two sorghum orthologous sequences (*Sb01g016775* and *Sb01g016700)*, one *Brachypodium distachyon* homolog (*Bradi4g45397)*, and 5 different *Tripsacum* species. The "*Lr34* orthologous cluster" is marked in red. The sequence from *Penicillium chrysogenum* (*Pc12g09900*) was used as outgroup to root the tree. Numbers indicate how many times the sequences to the right of the fork occurred in the same group out of 100 trees.
